# Experience-based virtual training system for knee arthroscopic inspection

**DOI:** 10.1186/1475-925X-12-63

**Published:** 2013-07-04

**Authors:** Shaw-Ruey Lyu, Yen-Kun Lin, Shian-Tang Huang, Hong-Tzong Yau

**Affiliations:** 1 Joint Center, Tzu-Chi Dalin General Hospital, Chia-yi, Taiwan, ROC; 2 Department of Mechanical Engineering, National Chung Cheng University, Chia-yi, Taiwan, ROC

## Abstract

**Background:**

Arthroscopic surgical training is inherently difficult due to limited visibility, reduced motion freedom and non-intuitive hand-eye coordination. Traditional training methods as well as virtual reality approach lack the direct guidance of an experienced physician.

**Methods:**

This paper presents an experience-based arthroscopic training simulator that integrates motion tracking with a haptic device to record and reproduce the complex trajectory of an arthroscopic inspection procedure. Optimal arthroscopic operations depend on much practice because the knee joint space is narrow and the anatomic structures are complex. The trajectory of the arthroscope from the experienced surgeon can be captured during the clinical treatment. Then a haptic device is used to guide the trainees in the virtual environment to follow the trajectory.

**Results:**

In this paper, an experiment for the eight subjects’ performance of arthroscopic inspection on the same simulator was done with and without the force guidance. The experiment reveals that most subjects’ performances are better after they repeated the same inspection five times. Furthermore, most subjects’ performances with the force guidance are better than those without the force guidance. In the experiment, the average error with the force guidance is 33.01% lower than that without the force guidance. The operation time with the force guidance is 14.95% less than that without the force guidance.

**Conclusions:**

We develop a novel virtual knee arthroscopic training system with virtual and haptic guidance. Compared to traditional VR training system that only has a single play-script based on a virtual model, the proposed system can track and reproduce real-life arthroscopic procedures and create a useful training database. From our experiment, the force guidance can efficiently shorten the learning curve of novice trainees. Through such system, novice trainees can efficiently develop required surgical skills by the virtual and haptic guidance from an experienced surgeon.

## Background

In recent years, arthroscopy has played a significant role for orthopaedic surgeons to perform minimally invasive surgery (MIS) on human joints. Compared to traditional open surgery, the MIS surgical technique provides benefits of less trauma, reduced pain, and faster healing. However, training of arthroscopic procedures is inherently difficult because of the limited visibility, reduced degrees of freedom of the instrument, and non-intuitive hand-eye coordination. It is vital for novice arthroscopic trainees to receive extensive and sufficient training before real surgeries to avoid surgical mistakes or unexpected injuries to patients. Conventional arthroscopic skill training relies on the use of cadavers, animals, or physical models. However, cadavers and animals cannot be repeatedly used and the physical model cannot provide realistic sensation feedback [1,2].

To overcome the above-mentioned shortcomings, virtual reality (VR) simulation provides an alternative solution [3-8]. A virtual model can be created from medical imaging data such as computed tomography (CT) or magnetic resonance imaging (MRI) and training can be performed in a VR immersed environment. A VR simulator can be reused many times and avoids risking patients’ health. Megali et al. [3] added training exercises to their already-developed navigation system. Results showed that when performing the training exercises, performance increases with surgical experience. Mabrey et al. [4] devised a virtual reality arthroscopic knee simulator consisting of a video display, two force feedback devices (one for the tool, one for the arthroscope), and a surrogate leg model with built-in sensors. However, they noted that hardware costs were high, with initial software development costs even higher. Heng et al. [5] built a virtual reality training system for arthroscopic knee surgery. They also developed a specific two-hand haptic device and put it into a box to present user with force feedback in a purely virtual environment. In their system, inspection training is supported and then the user could explore the virtual knee joint. In a similar way, Bayonat et al. [6] developed a virtual shoulder arthroscopy training simulator with force feedback. Moody et al. [7] presented a training environment that incorporated a realistic manipulable leg model to provide tactile augmentation to their virtual training environment. Again, virtual and external views of the arthroscope were shown. Tuijthof et al. [8,9] developed a physical environment to practice arthroscopic surgical skills to simulate operative real-life treatment. Y. Wang [10] developed a surgical procedure simulation system called vKASS for arthroscopic anterior cruciate ligament (ACL) reconstruction. Compared to previous studies, this system built a complete simulation for the entire procedure of arthroscopic ACL reconstruction. Recently, two commercial systems, such as Insight ArthroVR [11] and ARTHRO Mentor™ [12], can help trainees practice created scenarios and get force feedback by SensAble Omni®. Furthermore, they also provide a set of performance metrics for the objective assessment of skills development and the learning process at the end of each session.

Although the VR arthroscopic system can be used many times and a systematic training program can be embedded into the VR environment to replace conventional training methods, most of the current systems still have the following drawbacks. First, the training program is mostly based on a single play-script and a virtual model, which means it is not integrated with real-life clinical treatments. Real surgical trajectories cannot be captured and embedded into the system to match with different clinical situations. Second, traditionally, the rendering of a VR model from CT or MRI normally was monochrome. As a result, experienced surgeons still find it difficult to pass on their surgical skills to residents or novices with a VR-based training system. Without good guidance and practice, medical students cannot develop mature skills for such delicate task. Therefore, currently most VR-based training systems improve their rendering by texture mapping to strength their visual guidance. Study even showed that poor manipulation of the arthroscopic instrument can result in harmful collisions in real clinical situations [6]. Hence, the force guidance is a good choice to overcome these situations. Various studies [13-18] have shown that force guidance can effectively shorten the learning curves in many fields.

Moreover, most systems are costly due to expensive system integration of complex hardware and software components. Last but not the least, it is important to objectively evaluate or assess the skill levels of the trainees so that they can continuously improve their surgical skills. However, since real-life surgical case is difficult to be reproduced and incorporated into the traditional VR-based system, it is also hard to set up an objective evaluation standard to assess the arthroscopic surgical skills.

Currently, computer-aided surgical navigation systems are available to assist surgeons in different treatments [19-21]. In these systems, surgical planning, camera calibration, registration and motion tracking are combined. The use of the tracking technology continuously registers the position of patient and surgical instruments using different methods [22]. The magnetic, optical and vision-based tracking techniques are well-known. Generally, the cameras suffer from a distortion and then the perspective matrix is obtained using intrinsic and extrinsic matrix. These can be achieved using well-established camera calibration techniques [23,24].

During the treatment, the trajectory of the surgical tool operated by a surgeon can be recorded by the motion tracking. As a result, the clinical trajectory from an experienced surgeon can be integrated into the traditional VR-based training. Through the integration, the training system provides different real-life surgical cases and helps novices follow the clinical trajectory from the experienced surgeon. This paper presents an experience-based haptic training system for knee arthroscopic inspection. The key element of the proposed system is the integration of a motion tracking module with a haptic device such that the real-life surgical procedure and inspection trajectory can be captured and used to guide a novice trainee to repeat and practice the same clinical routine. Compared to traditional VR training system that has a single play-script based on a virtual model, the proposed system can capture and store different real-life clinical arthroscopic procedures and create a useful training database. Experienced surgeons can record their arthroscopic procedures in clinical treatment and pass on their surgical skills and precious experiences.

## Methods

**Figure 1:**
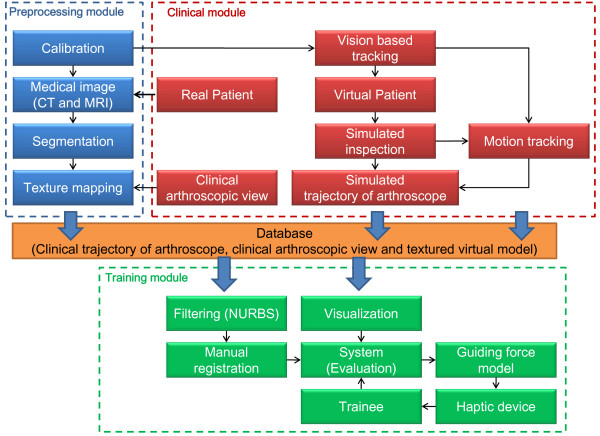
The system architecture.

The overall system consists of a pre-processing module, a clinical module and a training module, as shown in Figure 1. In the pre-processing module, a calibration needs to be done for a vision based tracking. Then, a virtual knee-joint model is built from a real patient’s medical images. The compartments are segmented from the virtual knee-joint model. In this paper, instead of using monotone shading or artificial texture mapping, we proposed to map the original images from the arthroscope to the reconstructed triangular surfaces from CT so that the model for the training will not be just a single-script example. This is important because some damaged or inflammation areas can not be displayed without color images. Hence, the 2D clinical arthroscopic view from clinical treatment is mapped to the 3D surface of the virtual knee-joint compartments. In the clinical module, the motion tracking is utilized to compute and record the arthroscopic procedure or inspection trajectory during a virtual surgical case performed by an experienced surgeon. The arthroscopic procedure is operated by an experienced surgeon and one fiducial marker is fixed on the arthroscope for the motion tracking. During the simulated inspection, a calibrated vision based tracking is used for the motion tracking. Thus, the simulated trajectory of the arthroscope including the position and orientation can be recorded. After the pre-processing and clinical inspection, the simulated trajectory of the arthroscope, the clinical arthroscopic view and its texture data can be integrated with the virtual knee-joint model to create a real-world clinical database. Finally, the trainees can select different scenarios from the database to practice their skills during the training module. To filter unwanted noise in the trajectory data, NURBS (Non-Uniform Rational B-Spline) interpolation is used to provide smooth trajectory of the arthroscope. Then, a registration procedure between the trajectory and the virtual knee-joint compartments is performed manually to match their positions and orientations in space. Using the tracking module, the realistic knee-joint model can be loaded from the database for visualization. The trainees not only can see the trajectory of the arthroscope in the realistic environment, but also operate a haptic device as the arthroscope. During the operation, the trainees can feel a guiding force if their operation is far from the original trajectory. Finally, we evaluate the trainees’ performances against the original trajectory with and without force guidance.

### Pre-processing module

#### Camera calibration

For vision based tracking, camera calibration is important to obtain good tracking accuracy before the motion tracking. In this system, a two-step calibration method [25] is adopted to reduce the distortion and compute the camera calibration parameters. The first step, distortion calibration, is used to solve the uneven spacing between the dots in the camera image. On the other hand, the second step of the camera calibration is for the computation of the camera intrinsic matrix, which consists of focal length, image center and effective pixel size to perform the perspective transformation for the camera.

#### Segmentation of medical images and texture mapping

**Figure 2:**
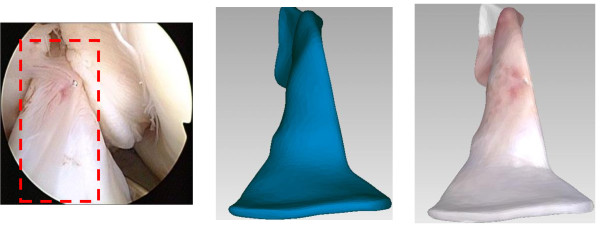
**The texture mapping for the anterior cruciate ligament. (a)** The clinical arthroscopic view, **(b)** The virtual ligament model, **(c)** The textured model.

To build the virtual knee-joint compartments, a medical image segmentation software (MIMICS, Leuven, Belgium) is used to segment the meniscus, articular cartilage, plica, ligament, femur, tibia, patella and fibula from medical images. After the segmentation, there are still unnecessary meshes such as holes and self-intersection in the virtual model. Hence, a scanning software (Geomagic Studio®, Morrisville, USA) is used to repair these unnecessary meshes and then carry out texture mapping. In the proposed system, the texture is from the clinical arthroscopic view and it is mapped to the surface of the virtual knee-joint compartments. After the texture mapping, finer surface details can be observed from these virtual compartments, as shown in Figure 2. Damaged or inflammatory surface areas can also be revealed after the texture mapping.

#### Motion tracking

**Figure 3:**
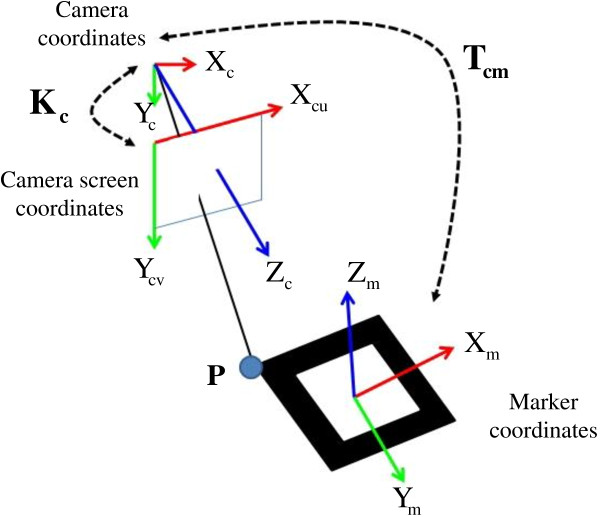
The relationship between camera, camera screen and marker coordinates.

In the motion tracking, a transformation, called camera extrinsic matrix (**T**_**cm**_), from the camera coordinates to the marker coordinates can be computed and recorded when the marker is detected. In this procedure, a camera intrinsic matrix (**K**_**c**_) is required to represent the relationship between the camera coordinates and camera screen coordinates. The main reason is that the marker detection is in the camera screen coordinates. The transformation among the camera, camera screen and marker coordinates can be illustrated in Figure 3.

**K**_**c**_ can be computed by the two-step camera calibration method and it only needs to be done once before the motion tracking. Once **K**_**c**_ is obtained, **T**_**cm**_ can be computed and recorded continuously. In Figure 3, a 3D point ***P*** in the marker and camera coordinates are separately ***P***_**m**_?=?[*x*_*m*_, *y*_*m*_, *z*_*m*_]^T^ and **Pc**?=?[*x*_*c*_, *y*_*c*_, *z*_*c*_]^T^. The relationship is as follows:

(1) $$P_{c}=\left[\begin{array}{c} x_{c}\\ y_{c}\\ z_{c}\\ 1 \end{array}\right]={\left[\begin{array}{cc} R_{3\times 3} & t_{3\times 1}\\ \begin{array}{ccc} 0 & 0 & 0 \end{array} & 1 \end{array}\right]}_{4\times 4}\left[\begin{array}{c} x_{m}\\ y_{m}\\ z_{m}\\ 1 \end{array}\right]=T_{\mathrm{cm}}P_{m}$$

where ***R***_3×3_ and ***t***_3×1_ are the rotation matrix and translation vector in **T**_**cm**_. In this paper, a square marker with a known-size is used as a base of the marker coordinates. Then, the camera extrinsic and intrinsic matrices can be computed by the Kato’s method [24]. During the motion tracking, the first stage is the marker detection in camera screen coordinates. Hence, an image analysis in camera screen coordinates is required. The image analysis includes building the binary image and identifying the black marker frame and symbol. Finally, the position and orientation for the marker can be obtained by the known **K**_**c**_ and the above information.

**Figure 4:**
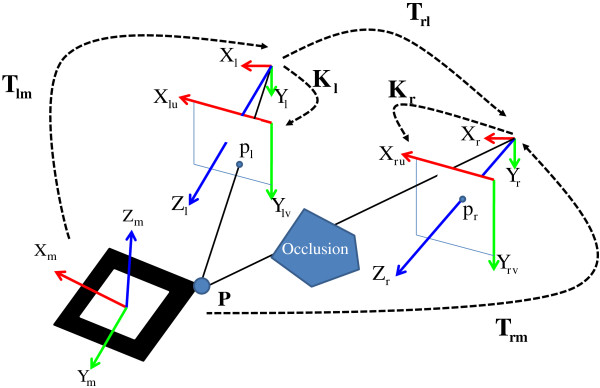
The illustration of the virtual world components and transformations in two cameras.

In the proposed system, two cameras are used to overcome the occlusion problem. Thus, the associated coordinate systems and the transformations in the virtual world are illustrated in Figure 4. All the coordinate systems used in this paper are right-handed. In the virtual world, the key references are the marker coordinates {*X*_*m*_,*Y*_*m*_,*Z*_*m*_}, the left tracking camera coordinates {*X*_*l*_,*Y*_*l*_,*Z*_*l*_}, the right tracking camera coordinates {*X*_*r*_,*Y*_*r*_,*Z*_*r*_}, the left tracking camera screen coordinates {*X*_*lu*_,*Y*_*lv*_}, and the right tracking camera screen coordinates {*X*_*ru*_,*Y*_*rv*_}.

Given a 2D projection *p*_*l*_ on the viewing plane of the left tracking camera screen coordinates, its 3D point ***P***_**l**_ in the left tracking camera coordinates can be computed by **K**_**l**_. Then, the **T**_**lm**_^-1^ times ***P***_**l**_ makes ***P***_**m***.*_ Once ***P***_**m**_ is obtained, its 3D projection ***P***_**r**_ in the right tracking camera coordinates can be computed by **T**_**rl**_. Its 3D relation between ***P***_**l**_ and ***P***_**r**_ is given by

(2) $$\left\{\begin{array}{c} P=T_{\mathrm{mr}}P_{r}=T_{\mathrm{ml}}P_{l}\\ P_{r}=T_{\mathrm{mr}}{}^{‐1}T_{\mathrm{ml}}P_{l}=T_{\mathrm{rl}}P_{l}\\ T_{\mathrm{rl}}=T_{\mathrm{mr}}{}^{‐1}T_{\mathrm{ml}} \end{array}\right)$$

**Figure 5:**
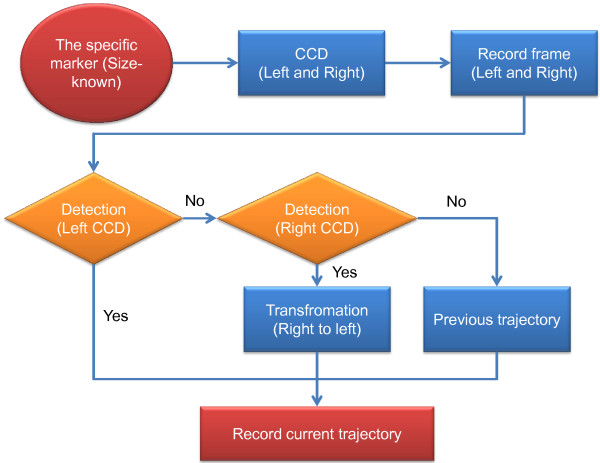
The flowchart of the motion tracking.

The flowchart for computing the trajectory is shown in Figure 5. Initially, there is a specific marker whose size is known and there are two CCDs (Charge-Coupled Device) in the proposed system. The proposed system can record the left and right frames in 30 frames per second at the same time. Then, the specific marker is used to detect the marker in these frames. During the detection for the specific marker, there are three kinds of conditions. First, the marker is detected in the left CCD frame and then the current trajectory can be obtained. Second, if the marker is not detected in the left CCD frame, the other detection in the right CCD frame can be executed to find the specific marker. If the marker is detected, the current trajectory computed can also be obtained by the transformation from Eq. (2). Third, if the marker cannot be detected from the left and right CCD frames, the current trajectory will be replaced by the previous trajectory. Finally, all trajectories in all frames can be recorded.

### Filtering of tracking data by NURBS

There are inherent measurement noises in the tracking data of the arthroscope. It is not desirable since a smooth trajectory is required for the arthroscopic training. In this paper, we adopt the use of NURBS fitting to the tracking data to generate smooth trajectory curves. It also helps compress the large amount of tracking data and provides efficient data storage. A *p*^th^-degree NURBS curve [26] is defined by.

(3) $$C\left(u\right)=\frac{\sum_{i=0}^{n}N_{i,p}\left(u\right)w_{i}P_{i}}{\sum_{i=0}^{n}N_{i,p}\left(u\right)w_{i}}=\sum_{i=0}^{n}R_{i,p}\left(u\right)P_{i}\;0\leq u\leq 1$$

where **P**_*i*_ are the control points, *w*_*i*_ the weights, and *N*_*i,p*_ the *i*^th^ B-spline basis function of the *p*^th^-degree defined on the non-periodic knot vector ***U***.

(4) $$U=\left\{\underset{p+1}{\underbrace{0,0,\cdots ,0}},u_{p+1},\cdots ,u_{n},\underset{p+1}{\underbrace{1,1,\cdots ,1}}\right\}$$

*N*_*i,p*_ is defined by Eqs. (5)-(6) and *R*_*i,p*_ the rational basis function, is defined by Eq. (7).

(5) $$N_{i,o}\left(u\right)=\left\{\begin{array}{c} 1\\ 0 \end{array}\;\begin{array}{c} \mathrm{if}\;u_{i}\leq u<u_{i+1}\\ \mathrm{otherwise} \end{array}\right)$$

(6) $$N_{i,p}\left(u\right)=\frac{u-u_{i}}{u_{i+p}-u_{i}}N_{i,p-1}\left(u\right)+\frac{u_{i+p+1}-u}{u_{i+p+1}-u_{i+1}}N_{i+1,p-1}\left(u\right)$$

(7) $$R_{i,p}\left(u\right)=\frac{N_{i,p}\left(u\right)w_{i}}{\sum_{j=0}^{n}N_{j,p}\left(u\right)w_{j}}$$

**Figure 6:**
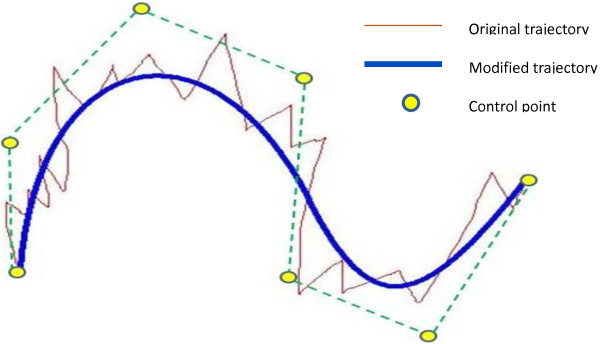
The NURBS curve.

Given an arthroscopic trajectory data with *n* discrete points {**Q**_*i*_|*i*?=?0,1,…*n*-1}, a NURBS curve which consists of *m* control points {**P**_*j*_|*j*?=?0,1,…,*m*-1} (*m*?<?*n*) can be constructed using Eq. (8) [27].

(8) $${\left[\begin{array}{ccccc} 1 & 0 & 0 & \cdots & 0\\ R_{0,p}\left({\bar{u}}_{1}\right) & R_{1,p}\left({\bar{u}}_{1}\right) & R_{2,p}\left({\bar{u}}_{1}\right) & \cdots & R_{m-1,p}\left({\bar{u}}_{1}\right)\\ R_{0,p}\left({\bar{u}}_{2}\right) & R_{1,p}\left({\bar{u}}_{2}\right) & R_{2,p}\left({\bar{u}}_{2}\right) & \cdots & R_{m-1,p}\left({\bar{u}}_{1}\right)\\ \vdots & \vdots & \vdots & \ddots & \vdots \\ R_{0,p}\left({\bar{u}}_{i}\right) & R_{1,p}\left({\bar{u}}_{i}\right) & R_{2,p}\left({\bar{u}}_{i}\right) & \cdots & R_{m-1,p}\left({\bar{u}}_{i}\right)\\ 0 & 0 & 0 & \cdots & 1 \end{array}\right]}_{\mathrm{nxm}}{\left[\begin{array}{c} P_{0}\\ P_{1}\\ P_{2}\\ \vdots \\ P_{i}\\ P_{m-1} \end{array}\right]}_{\mathrm{nxm}}={\left[\begin{array}{c} Q_{0}\\ Q_{1}\\ Q_{2}\\ \vdots \\ Q_{i}\\ Q_{m-1} \end{array}\right]}_{n\times 1}m<n$$

where the parameter ${\bar{u}}_{i}$ corresponding to the tracking data points **Q**_*i*_ can be calculated as

(9) $${\bar{u}}_{i}=\frac{t_{i}-t_{0}}{t_{1}-t_{0}}$$

where *t*_0_ is the tracking start time, *t*_1_ the tracking end time, and *t*_i_ the time corresponding to **Q**_*i*_. An example is described in Figure 6.

After the trajectory of the arthroscopic inspection is captured, the tracking data is filtered by NURBS. Finally, the trajectory is registered to the virtual model created from CT data.

### Force guidance of inspection trajectory

**Figure 7:**
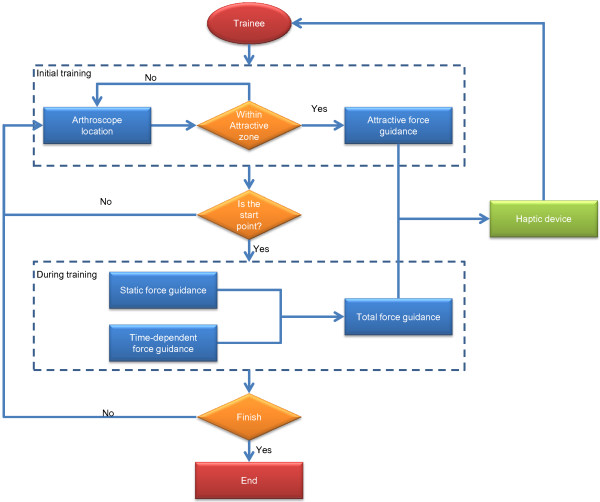
The flowchart of force guidance for trainees’ movements.

After obtaining the trajectory of the arthroscope, trainees can follow the optimal inspection path from experienced surgeons according to the clinically recorded trajectory. In this system, trainees not only can see the trajectory in the virtual environment, but they can also be guided by haptic force feedback. In order to provide force feedback, a haptic device (Phantom, SensAble Technologies, Wilmington, MA) is used for the creation of the guiding force. The guiding force can provide guiding direction, prevent the virtual arthroscope from leaving the original trajectory, and assist the trainees in performing the surgical simulation. There are three guiding forces including attractive force, static force and time-dependent force in the proposed simulator. The overall flowchart of the guiding force in the proposed simulation is illustrated in Figure 7.

#### Initialization with attractive force

**Figure 8:**
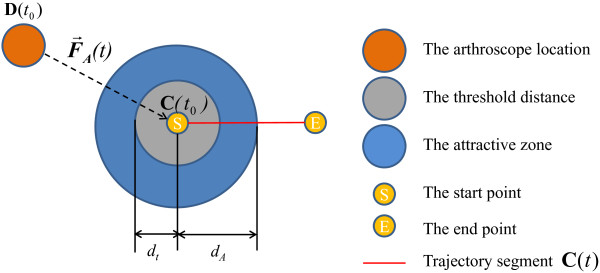
The attractive force guidance.

**Figure 9:**
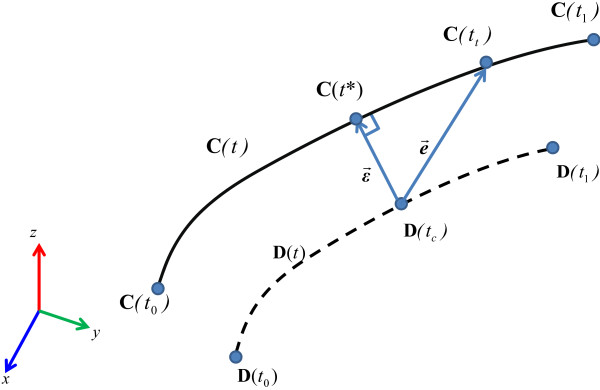
The static and time-dependent force guidance.

A recorded trajectory can be represented by a NURBS curve **C**(*t*), in which t is the recorded time parameter.

(10) $$t=t_{0}+u\left(t_{1}-t_{0}\right)$$

and *u* is the normalized NURBS parameter between 0 and 1. The trainee will be guided by the haptic device to follow the trajectory curve **C**(*t*) which simulates the nominal inspection path obtained from the experienced surgeon. A guiding force is needed for the trainee to follow or track **C**(*t*). Assuming the trainee’s trajectory **D**(*t*) deviates with some distance from **C**(*t*), we can provide an attraction force in the initialization stage to attract **D**(*t*_0_) to **C**(*t*_0_). Figure 8 represents the attractive force ${\vec{F}}_{A}\left(t\right)$ in a segment of the trajectory curve. When the distance between **C**(*t*) and **D**(*t*_0_) is larger than *d*_*A*_, it is visually guided by the VR simulation and the haptic force ${\vec{F}}_{A}\left(t\right)$ to help the trainee move the arthroscope towards the target **C**(*t*_0_) (see Figure 9).

(11) $${\vec{F}}_{A}\left(t\right)=k_{A}d_{A}\frac{C\left(t_{0}\right)-D\left(t\right)}{\left|C\left(t_{0}\right)-D\left(t\right)\right|}$$

But when the arthroscope probe head comes between the sphere of radius *d*_*t*_ and *d*_*A*_, the attractive force will be reduced linearly following Eq. (12).

(12) $${\vec{F}}_{A}\left(t\right)=k_{A}\left(C\left(t_{0}\right)‐D\left(t\right)\right)$$

When the arthroscope probe head comes within the sphere of radius *d*_*t*_, the attractive force is zero.

#### Static force guidance

After the initialization, the trainee will be guided by force to follow the trajectory **C**(*t*). If we only consider the desired trajectory **C**(*t*) as a static curve without considering the time constraint the trainee needs to follow and finish the arthroscopic inspection, then we only need to consider the contour error $\vec{\varepsilon }$ between **C**(t*) and **D**(*t*). The projected **C**(t*) will become the current target point.

(13) $$\vec{\varepsilon }=C\left(t*\right)-D\left(t\right)$$

Hence, the guiding force is defined as the static (normal) guiding force ${\vec{F}}_{s}$.

(14) $${\vec{F}}_{s}\left(t\right)=k_{s}\vec{\varepsilon }=k_{s}\left(C\left(t*\right)-D\left(t\right)\right)$$

For novice trainees, it means there is no time constraint or pressure to push the trainee in finishing the inspection procedure. The trainee can follow his own speed with which he is comfortable. The static or normal force will guide the arthroscope in getting close to the recorded trajectory in the normal direction. However, the trainee can still be visually guided by the VR environment to move along the path.

#### Time-dependent force guidance

In this study, it is assumed the recorded trajectory by the experienced surgeon is the standard inspection path to follow. This includes not only the geometric path, but also the various speed throughout the trajectory. An experienced surgeon knows where to move the probe head faster, and where to slow it down for better observation and collision avoidance. Therefore, for advanced and realistic force guidance, **C**(*t*) is a time-dependent trajectory to follow, as depicted in Figure 9. The time-dependent guiding force is ${\vec{F}}_{d}$, which can be considered as the combination of normal and tangential guiding force.

(15) $${\vec{F}}_{d}\left(t\right)=k_{d}\vec{e}=k_{d}{\left[e_{x},e_{y},e_{z}\right]}^{T}$$

where the tracking error $\vec{e}$ is the vector between the target point **C**(*t*_*t*_) and the current point **D**(*t*_*c*_). Furthermore, as the trainee obtains more experiences by repeated practices, the force guidance can be reduced and eventually removed. This can be easily implemented by the following scheme.

(16) $$\vec{F}=\left(1-\nu \right)\cdot k\cdot \vec{e}\;0<\nu <1$$

where *v* is the training strength parameter. When *v*?=?0, there is full force guidance; when *v*?=?1, the guidance is completely removed.

## Results and discussion

The hardware of our system includes a haptic device, a personal computer and a display monitor. The Phantom haptic device has 6-DOF manipulation and provides 3-DOF (x,y,z) force feedback in the guiding module. Our system is executed on a Pentium 4 1.5 GHz PC equipped with NVidia GeForce3 graphics card. The PC handles all computation consisting of guiding model and visual rendering. The proposed system is implemented in OpenGL and C++.

**Figure 10:**
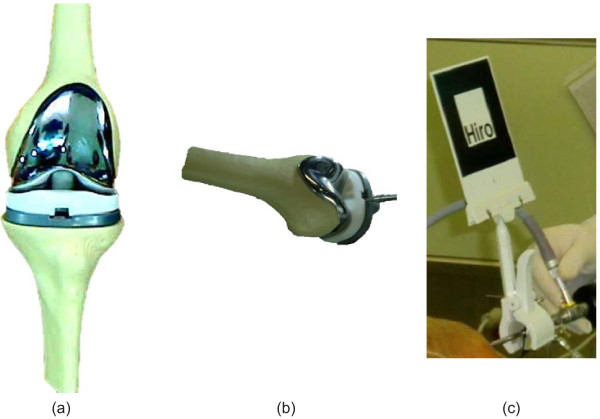
**The simulated inspection. (a)** The real knee model, **(b)** The simulated inspection, **(c)** The fiducial marker is fixed on the arthroscope.

In the clinical module, the experienced surgeon operated an arthroscope in a virtual patient’s knee during the simulated inspection, as shown in Figure 10(a) and (b). One fiducial marker made of acrylic was disinfected before inspection and fixed on the arthroscope, as depicted in Figure 10(c). Finally, the trajectory of the arthroscope can be saved into the database.

### Virtualization

**Figure 11:**
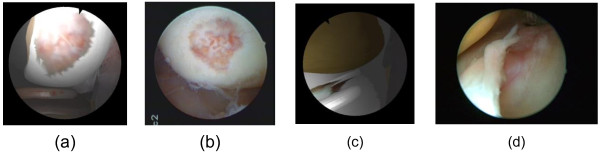
**Visualization for real and virtual arthroscopic view. (a)** Virtual inflammatory articular cartilage, **(b)** Real inflammatory articular cartilage, **(c)** Virtual inflammatory medial meniscus, **(d)** Real inflammatory medial meniscus.

In the proposed system, a virtual knee-joint model can be obtained from the database. The original surface of the virtual knee-joint model was black and white. However, it is now enriched with color detail in the visualization because of the texture mapping, as indicated in Figure 11.

### Filtering of tracking data by NURBS

**Figure 12:**
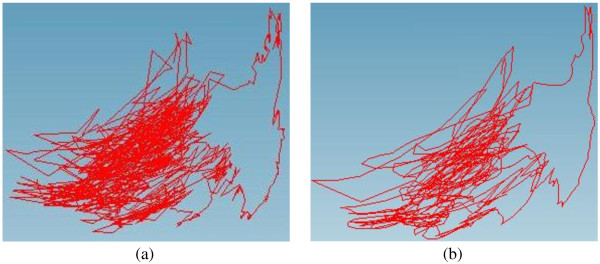
**Trajectory reconstruction with NURBS curve. (a)** Original trajectory, **(b)** Modified trajectory.

After the virtual knee-joint model is loaded, the previous trajectory of the arthroscope for the inspection is also retrieved from the database. The original trajectory has been filtered by NURBS and produced much smoother trajectory curves. The noises in the original trajectory of the arthroscope were effectively filtered and the amount of data was much compressed (see Figure 12).

### User interface

**Figure 13:**
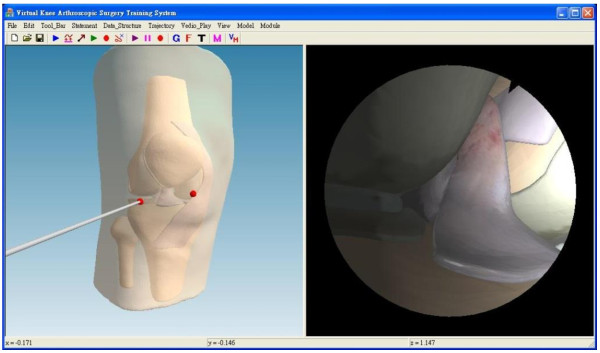
The user interface: The left window is external viewpoint window and the right window is arthroscopic viewpoint window (The red points are the insertions for the arthroscope).

The clinical scenario is displayed on a LCD panel, which contains two separate windows, as shown in Figure 13 (Additional file 1). The external viewpoint window displays the surgical scene as viewed form an external viewpoint such as surgeon’s view. This view can be adjusted depending on the surgeon’s preference by changing the camera position, orientation and magnification. The arthroscopic viewpoint window can display the recorded clinical view, as well as the virtual view. Although the virtual view is created by simulation, the clinical arthroscopic view is mapped onto the surfaces of the virtual knee-joint compartments in the virtual view. The trainees can switch between these two views if the patient’s medical images are previously obtained. Otherwise, the trainees can only see the virtual view.

### Force guidance of inspection trajectory

**Figure 14:**
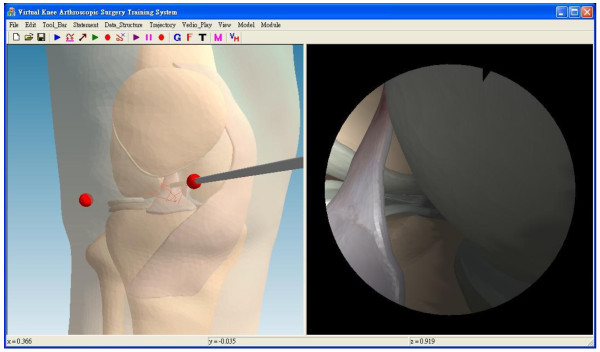
The visualization of the trajectory.

**Figure 15:**
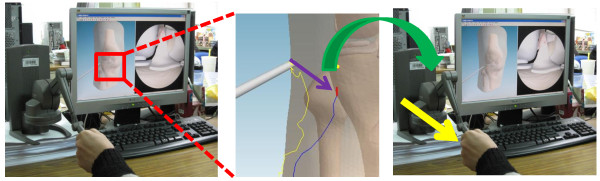
Force Guidance of Inspection Trajectory.

The trainees can see the trajectory in the proposed system as shown in Figure 14. Then, users can feel the force guidance by the haptic device when their virtual probe head deviates from the planned trajectory, as indicated in Figure 15.

### Evaluation

**Figure 16:**
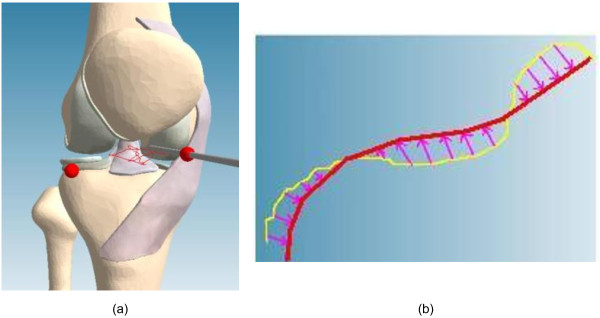
**The training trajectory and error. (a)** 3-D motion, **(b)** The path error.

In this paper, we evaluate that the force guidance improves the training for inspection of the knee. Thus, eight participants perform an inspection of the knee whose trajectory is obtained from an experienced surgeon in the clinical module. All participants were right-handed and they operated the same inspection with and without force guidance. To evaluate participants’ performance, the normal path error and operating time were recorded. The normal path error was based on the trajectory from experienced surgeon in clinical module and the movements of the Phantom tip operated by each participant. A 3D motion of the ideal and experimental trajectory is illustrated in Figure 16(a) (Additional file 2). Then, the normal path error is the shortest distance between the trajectory from the trainee and the trajectory from the experienced surgeon, as shown in Figure 16(b).

To avoid differences in performance related to unease with the instruments, they used initially the haptic device to perform some basic operations without force guidance in the VR environment [28]. These basic operations were based on the manufacturer established settings, and were not modified by authors. After achieving familiarity with the haptic device and basic operations, the participants completed the first inspection without force guidance and recorded their first operating time and the normal path error as first data. Then, they repeated the same inspection five times without recording for practice. After that, they did the inspection again and recorded their operating time and the normal path error as the second data. Thus, we can understand the learning curve after continuous practices five times according to the first and second data.

In order to avoid the effect of accumulated learning experience, at least 3 days were allowed to pass after the completion of the inspection without the force guidance before we started the new inspection section with the force guidance. The participants performed the inspection with force guidance in the same manner as before. Data on the completion time and error were described in Tables 1 and 2.

**Table 1:** Without the force guidance

	**A**	**B**	**C**	**D**	**E**	**F**	**G**	**H**
AVG. 1 (cm)	0.288	0.7	0.204	0.476	0.331	0.541	0.378	1.525
SD. 1	0.13	0.37	0.136	0.21	0.134	0.247	0.183	1.059
Time (Second)	24.914	13.923	28.855	13.895	15.995	14.222	16.623	30.095
AVG. 2 (cm)	0.183	0.45	0.447	0.383	0.212	0.261	0.219	0.545
SD. 2	0.168	0.35	0.24	0.181	0.145	0.183	0.155	0.325
Time (Second)	13.418	13.745	14.059	9.859	15.232	7.323	15.614	12.041

**Table 2:** With the force guidance

	**A**	**B**	**C**	**D**	**E**	**F**	**G**	**H**
AVG. 1 (cm)	0.168	0.109	0.575	0.248	0.1	0.374	0.378	0.135
SD. 1	0.094	0.078	0.24	0.091	0.094	0.197	0.293	0.067
Time (Second)	13.99	11.195	15.259	9.055	19.964	9.286	9.205	9.668
AVG. 2 (cm)	0.107	0.482	0.357	0.234	0.173	0.187	0.116	0.126
SD. 2	0.065	0.251	0.16	0.134	0.093	0.085	0.113	0.079
Time (Second)	11.114	13.159	8.1	9.232	13.077	8.455	11.782	8.891

**Figure 17:**
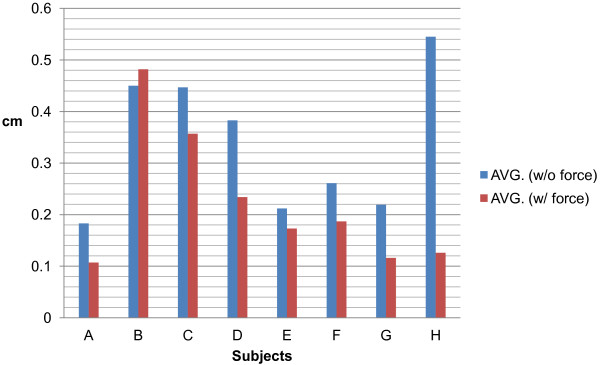
The subject’s performance of arthroscopic inspection on the same simulator was done with and without the force guidance.

The experiment reveals that most participants’ performances are better after they repeated continuously the same inspection five times. The standard deviations are also clear indications to show that they performed better after repeating the same operation. Furthermore, we should notice that most participants’ performances with the force guidance are better than those without the force guidance, as shown in Figure 17. The performance includes the mean normal path error, standard deviation and the operating time. In the experiment, the average error with the force guidance is 33.01% lower than that without the force guidance. The operating time with the force guidance is 14.95% less than that without the force guidance. Even though B’s second mean normal path error is larger than the first one in the haptics-enhanced inspection, his second standard deviation is similar to the first one. It indicates that there were few tremors during B’s operation. Data on the normal path error showed a trend toward a benefit from the force guidance in the inspection.

**Figure 18:**
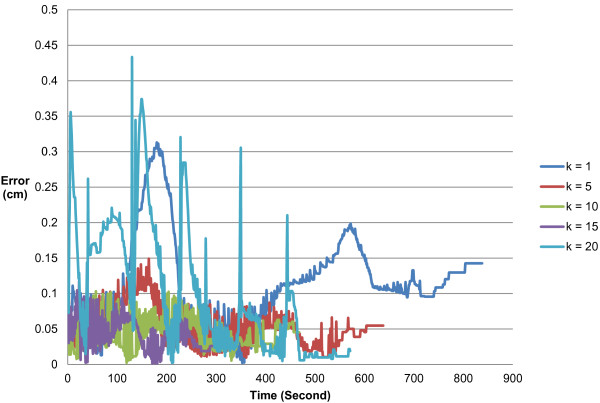
The effect for the magnitude of force.

The force guidance is important to shorten the learning time. Hence, the magnitude of force has played an important role in the force guidance. The magnitude of force in this system can be adjusted by the simulated spring coefficient. Figure 18 indicates that the higher the spring coefficient, the better the learning result. However, unstable force will occur if the spring coefficient is set too high.

## Conclusions

In this paper, we have discussed the motivations for developing a novel virtual arthroscopic training system. The system focuses on the experience-based inspection of anatomic knee structures. In this system, the trajectory of the arthroscope from an experienced surgeon can be recorded by vision tracking and built into a database. Then, the noise of the trajectory can be filtered by NURBS. Data compression advantage is also achieved. After obtaining the NURBS curve, it can be used to review the entire inspection procedure and guide the trainees in the virtual environment. Furthermore, the clinical arthroscopic view is mapped to the surface of the virtual knee-joint compartments. Hence, more realistic environment can be visualized. Then, the trainee can operate the haptic device along the NURBS trajectory during the force guidance. The trainees will feel the force guidance when their virtual arthroscope probe head deviates from the planned trajectory. The experimental results suggest that stronger educational effect can be achieved by force guidance for novice trainees. Currently, the deformation of the soft tissue has not been implemented in the system. In the future, we will develop the soft tissue deformation model to strength the simulation effect. In addition, a specific haptic device will be built to increase the DOF to simulate the practical treatment. Finally, a multi-metric scoring system will be integrated into the proposed system to objectively evaluate the users’ performances.

## Abbreviations

VR: Virtual reality; MIS: Minimally invasive surgery; CT: Computed tomography; MRI: Magnetic resonance imaging; CCD: Charge-coupled device; NURBS: Non-uniform rational B-spline; AVG: Average; SD: Standard deviation.

## Competing interests

The authors declare that they have no competing interests.

## Authors’ contributions

SRL proposed conception, design and drafted the manuscript. YKL participated in its design and coordination and helped to draft the manuscript. STH participated in the design of the study and performed the analysis. HTY conceived of the study, and participated in coordination and drafted the manuscript. All authors read and approved the final manuscript.

## Supplementary Material

Additional file 1 **Virtual inspection [**http://youtu.be/lKplX2-lc34**].** Click here for file

Additional file 2 **Virtual Inspection with force guidance [**http://youtu.be/hEW0eaKGZP8**].** Click here for file
